# Development of a custom-made 2.8 T permanent-magnet dipole photon source for the ROCK beamline at SOLEIL

**DOI:** 10.1107/S1600577523002990

**Published:** 2023-05-10

**Authors:** Pascale Brunelle, Nicolas Béchu, Valérie Briois, Fabrice Marteau, Marc Ribbens, Philippe Berteaud, Xavier Delétoille, Eric Dupuy, Christian Herbeaux, Marie Labat, Alain Lestrade, Amor Nadji, Laurent Nadolski, Mohamed Nouna, Jean-Baptiste Pruvost

**Affiliations:** a Synchrotron SOLEIL, L’Orme des Merisiers, Départementale 128, 91190 Saint-Aubin, France; b Centre National de la Recherche Scientifique, UR1, France; University of Essex, United Kingdom

**Keywords:** SOLEIL, synchrotron light source, permanent magnet dipole, ROCK photon beamline

## Abstract

In August 2021, the SOLEIL storage ring was restarted after the summer shutdown with a new bending magnet made entirely of permanent magnets. Producing a magnetic field of 2.8 T, it replaced one of the 32 electromagnetic dipoles (magnetic field of 1.7 T) of the ring to allow the ROCK beamline to exploit more intense photon fluxes in the hard X-ray range, thus improving the time resolution performances of the beamline for experiments carried out above 20 keV. The reduction of the new dipole magnetic gap required to produce the higher field has led to the construction and installation of a new vacuum vessel. The realization of the new dipole with permanent magnets was a technological feat due to the very strong magnetic forces. The permanent-magnet assembly required dedicated tools to be designed and constructed. Thanks to accurate magnetic measurements, a precise modelization of the new dipole was performed to identify its effects on the electron beam dynamics. The first measurements carried out on the ROCK beamline have highlighted the expected increase in photon flux, and the operation performances remain unchanged for the other beamlines. Here, the major developments and results of this innovative project are described in terms of technology, electron beam dynamics and photon beam performance on the ROCK beamline.

## Abstract 2

Since August 2021, the French synchrotron light source SOLEIL has been running with a 1 m-long permanent-magnet dipole producing a magnetic field of 2.8 T. This is a major modification of the storage ring that allows the high-energy photon flux for the ROCK beamline to be increased and significantly improves the resolution for experiments performed above 20 keV.

## 1. Introduction

**Figure 1:**
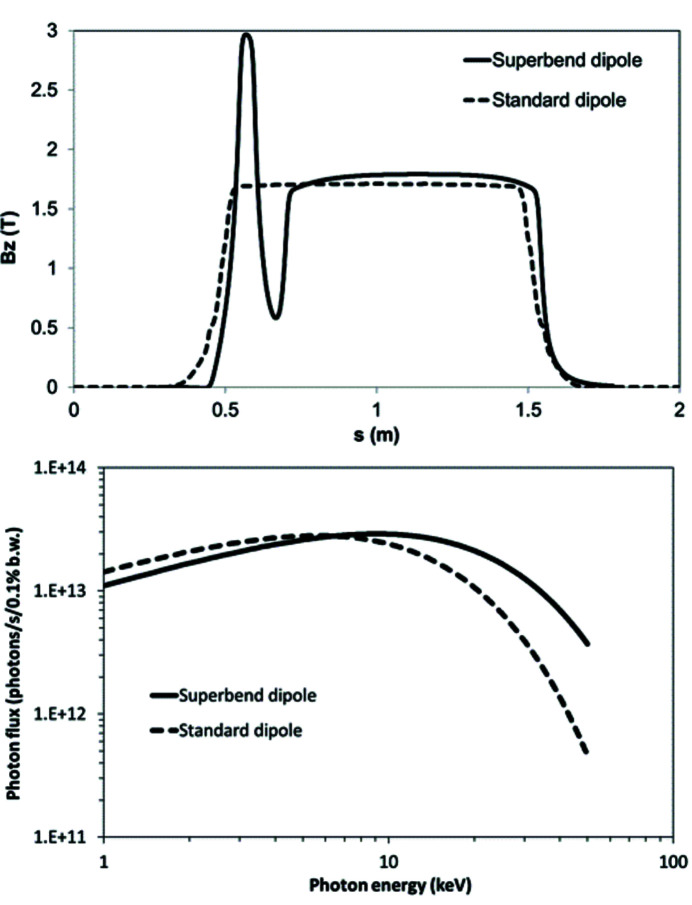
Comparison of the performance of the standard dipole and the Superbend. (Top) Variation of the magnetic field along the electron trajectory. (Bottom) Variation of the photon flux as a function of the photon energy (calculated at 8.7 m from the source point for an aperture of 13 mm × 2.4 mm).

The SOLEIL 2.75 GeV third-generation synchrotron light source delivers extremely stable, high-average-brightness photon beams to 29 beamlines, 20 on insertion devices and 9 on bending magnets, with photon energies in a range of ten orders of magnitude from the IR–UV–VUV up to hard X-rays (https://www.synchrotron-soleil.fr). Since 2006, the storage ring lattice, based on a modified double-bend achromat structure, has been modified to accommodate photon beamline requirements, and two major local modifications have been performed leading to a onefold symmetry (Nadolski *et al.*, 2021 ▸). In multi-bunch filling pattern operation (500 mA electron beam current), the average lifetime is 10 h (1% emittance coupling and 2.8 MV RF-voltage), and the injection efficiency is 80%. In 2017, a new photon source was requested for the ROCK beamline (https://www.synchrotron-soleil.fr/en/beamlines/rock), capable of producing a higher photon flux than the initial source between 20 and 40 keV, to substantially improve the signal-to-noise ratio (S/N) of X-ray absorption spectroscopy measurements in this energy range for a sub-second time resolution. This flux increase (a factor of 5 at 40 keV) is obtained by increasing the magnetic field at the source point of the photon beam from 1.7 T [standard dipole (Brunelle *et al.*, 2006 ▸)] to 2.8 T (Fig. 1 ▸). In 2018, after a feasibility study, it was decided to design and built a new dipole called Superbend to be installed in place of the existing ROCK standard dipole photon source. A first technological breakthrough was achieved with the choice of a permanent-magnet (PM) dipole to replace the standard electromagnetic dipole, making SOLEIL the first third-generation light source to operate with such a unique PM dipole photon source. Indeed, other third-generation synchrotron light sources in the world are running with Superbend-type dipoles but these are of the electromagnetic type, not the PM type, *e.g.* the ALS (Robin *et al.*, 2002 ▸) (5 T superconducting magnets) and SLS (2.9 T normal conducting magnets) (Gabard *et al.*, 2011 ▸) storage rings. The specificity of this project is that only one Superbend is installed in the ring whereas for the other machines the number of Superbends respects the symmetry of the magnetic structure. It is well known that breaking the symmetry of a storage ring inevitably degrades the robustness of the electron beam dynamics and leads to poorer performance (Loulergue *et al.*, 2010 ▸), which was a challenge for SOLEIL. Another specificity of the SOLEIL Superbend is the position of the source point at a deviation of 1°, very close to the dipole entry, whereas for the other machines the strong field is created in the middle of the dipole, again with a more advantageous symmetry. In addition, it was chosen to produce the high magnetic field of 2.8 T only at the ROCK photon source point, over a short portion of the beam path, avoiding shifting the beamline optics and instruments and keeping almost unchanged the power to be dissipated on the existing absorbers, allowing then to use the standard model for the new vacuum vessel and crotch. This choice also imposed the initial value of the magnetic field to not exceed 1.7 T elsewhere in the dipole to achieve the 11.25° standard beam trajectory deviation, corresponding to an integrated field of 1.793 T m. The 2.8 T value of the high magnetic field is the result of a compromise between the surface of the magnetic pole and the magnetic gap, the latter being directly related to the thickness of the vacuum vessel wall and the internal vertical aperture of the vacuum vessel allowed by the electron beam. The new dipole is made with a unique yoke, whose length is very close to that of the standard dipole. The magnetic field is longitudinally shared in two parts – the so-called high field part (HFP) and the low field part (LFP) (Fig. 1 ▸). The vertical inner aperture of the vacuum vessel was optimized in each of the low field and high field parts to leave the maximum free space for the electron beam, thus leading to different apertures in the two parts. The vertical profile was optimized so that no heating could degrade the quality of the permanent magnets.

Another challenge was the operation of the storage ring with such a strong and permanent magnetic field. Indeed, the high value of the magnetic field obtained with a reduced width of the magnetic pole generates a strong field roll-off in the electron beam stay-clear region, resulting in an additional sufficiently high sextupolar component that affects the transverse dynamics of the electrons, and consequently the beam lifetime and the injection efficiency. It was therefore essential to describe the new magnetic field in the lattice model as accurately as possible, which was possible thanks to the field mapping performed during the magnetic measurements.

This paper presents the requirements of the ROCK beamline that motivated this innovative project, the design and construction challenges that were met for the new dipole and the new vacuum vessel, the excellent quality of the magnetic measurements necessary to carry out the precise modeling of the effect of the new magnetic field on the electron beam dynamics, the commissioning of the new dipole together with the new vessel and, finally, the results of the first experiments carried out on the ROCK beamline with its new photon source.

## 2. Motivation for a new dipole

ROCK is the Quick-EXAFS photon beamline at SOLEIL funded in 2011 by the French Agency, Agence Nationale de la Recherche, in the framework of the ‘Investissements d’Avenir’ program (ANR-10-EQX-45-01) and in operation since 2015. The optical layout of the beamline has been tailored for sub-second time-resolved characterization of catalysts and energy-related materials (Briois *et al.*, 2016 ▸; La Fontaine *et al.*, 2020 ▸). A first toroidal collimating mirror, installed in the storage ring tunnel at a position as close as possible to the source (10.15 m) for maximizing its vertical acceptance, delivers in the optical hutch a pink beam with electromagnetic radiation covering the 4–42 keV energy range. Sub-second time-resolved measurements rely not only on the mechanical performances of the Quick-EXAFS monochromators for fast and continuous scanning of the photon energy but also on the availability of at least 10^11^ to 10^12^ photons s^−1^ at the sample position. Even if the ROCK Quick-EXAFS monochromators (Briois *et al.*, 2016 ▸; Fonda *et al.*, 2012 ▸) were designed for recording spectra with an oscillation frequency up to 20 Hz, *i.e.* 25 ms time resolution per spectrum, the 1.7 T standard dipole source with critical energy of 8.55 keV does not satisfy the second criterion of photon flux for energies above ∼25 keV (Briois *et al.*, 2016 ▸) leading to inherent loss of quality of the Quick-EXAFS data recorded at the high energy range covered by the beamline. The importance in catalysis and energy-related science of 4*d* transition metal elements with *K* absorption edges falling within 18–29.2 keV (Zr to Sn), of pnictogen Sb (*K*-edge at 30.5 keV), chalcogen Te (*K*-edge at 31.8 keV) and halogen I (*K*-edge at 33.2 keV) elements of row 5 of the periodic table and of the Ce 4*f* element (*K*-edge at 40.4 keV) gave a strong motivation to extend the optimal time resolution performance of the beamline to the high energy range by shifting the critical energy of the source by nearly 6 keV compared with the electromagnetic 1.7 T source.

## 3. Design and construction of the new dipole

The main parameters of the Superbend are listed in Table 1 ▸.

### 3.1. Magnetic design

**Figure 2:**
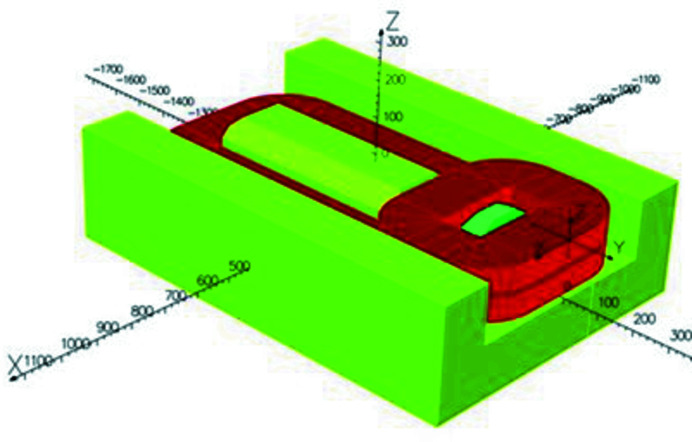
View of the bottom part of the electromagnetic version of the Superbend. (Green) The yoke and the poles. (Red) The coils. *X*, *Z* and *Y* are the horizontal, vertical and longitudinal dimensions, respectively, expressed in millimeters. This design was rejected and replaced by a permanent-magnet version.

**Figure 3:**
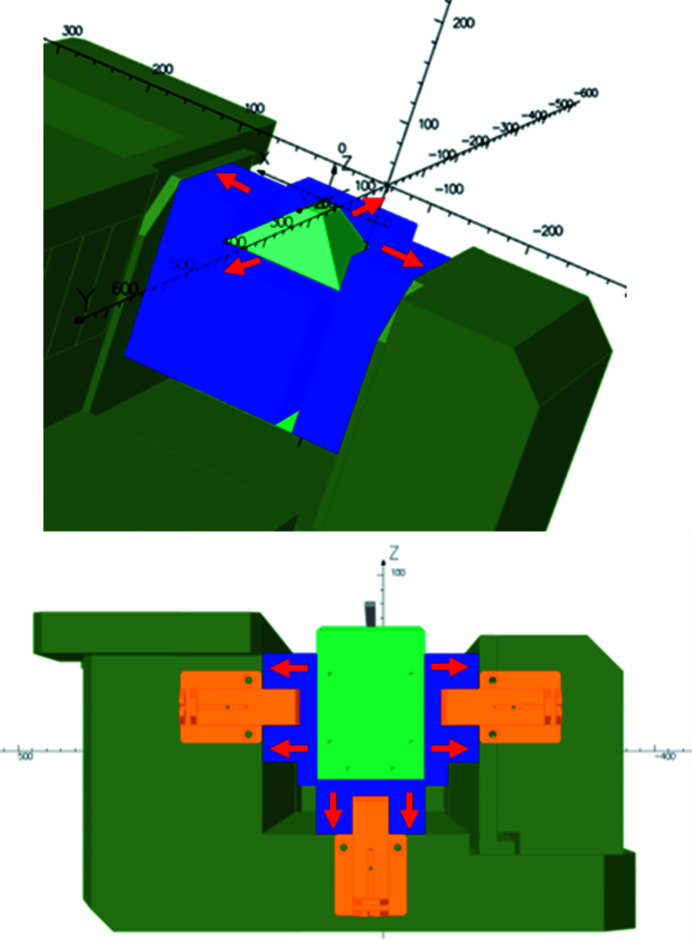
(Top) Partial view of the high field part (half-yoke) of the Superbend. (Bottom) Transverse view of the exit of the low field part (half-yoke) of the Superbend. (Light green) Pole. (Blue) PM blocks. (Dark green) Yoke. (Orange) Magnetic short circuit. (Red arrows) Direction of the magnetization of the PM blocks. *X*, *Z* and *Y* are the horizontal, vertical and longitudinal dimensions, respectively, expressed in millimeters.

The calculations were performed using the code *OPERA* (Dassault Systemes SIMULIA). The magnetic design went through a long process to determine the technology that would be used for the dipole. The first idea was to design an electromagnetic dipole with standard low-carbon steel yoke Fe–Co poles for the high field and coils made of hollow conductors (Fig. 2 ▸). It gave a dipole with a 500 A range current with a power consumption of about 37 kW. As a comparison, the standard electromagnetic dipole in the SOLEIL storage ring consumes about 12 kW. Since the modification of the hydraulic infrastructure in the storage ring tunnel requested for the installation of such a device turned out to be so important, this solution was rejected. Following this decision and knowing that Sirius, the new Brazilian source (Citadini *et al.*, 2018 ▸), made a successful PM dipole with a 3.2 T peak field, the PM solution was chosen. As mentioned previously, the dipole has two parts: the HFP, which allows the electron beam to produce the expected radiation for the ROCK beamline, and the LFP, which provides the integrated magnetic field required for the 11.25° bending (1.793 T m). The HFP comprises an iron–cobalt pyramidal pole where PMs are installed on each side. The LFP comprises a low-carbon steel pole where PMs are installed on each side and underneath (Fig. 3 ▸). Note that the size of the base of the strong field pole has been optimized with the priority of reaching the required field value for the ROCK beamline, taking into account the constraints on the gap imposed by the vacuum chamber mechanical robustness. The resulting very small size of the strong field pole tip generates a strong field roll-off and a strong sextupolar component (see Fig. 18 later). The pole has a pyramidal shape with a 120 mm × 120 mm square base to a 19.5 mm × 45 mm pole tip. This pyramidal shape is 48 mm high on top of the 145.5 mm pole block. It was not possible to significantly reduce the sextupolar component without reducing the high field value. A possible solution was to use the low field part to create an opposite integrated sextupolar component, but the curvature of the beam trajectory would have led to a complicated design to be realized.

To prevent any discrepancy between the specified field integral and the measured one, due to the sensitivity of the PMs to temperature variation, a set of dipolar coils are installed around the LFP to be able to adjust the field integral during the operation of the storage ring if necessary. These coils can be fed by a bipolar 100 A/600 W power supply with 15 turns and can vary the field integral by ±3%.

To adjust the field integral during magnetic measurements, some stainless-steel shims were inserted between the two yokes. These shims increase the gap between the poles and add an air gap in the yoke that modifies the integrated field. The designed air gap is 2.0 mm. The adjustment of the field integral by means of stainless shims is a long process in terms of manipulation of the dipole where the yoke has to be split into two to insert the shims. So, the dipole was also equipped with some magnetic short circuits (MSCs) on the exit side (Figs. 3 ▸ and 4 ▸). The MSCs consist of pieces of low carbon steel which can slide between the yoke and the pole in order to capture the flux from the PM and then induce a variation of the field integral. The PMs are made of NdFeB with a remanent field *B*_r_ of 1.38 T and a coercitive force *H*_cj_ of 1580 kA m^−1^. The requested size for the PM block is 120 mm × 60 mm × 60 mm. Due to some manufacturing limitations, the PM blocks are made of two 120 mm × 60 mm × 30 mm blocks and the magnetization is along the 30 mm dimension. Only one type of PM block is used for both the LFP and the HFP. A high value of the remanent field was required to reach the 2.8 T peak field value and to have a reasonable volume of PM materials. This was also requested for the low field part where the vertical gap is 36 mm.

### 3.2. Mechanical design

**Figure 4:**
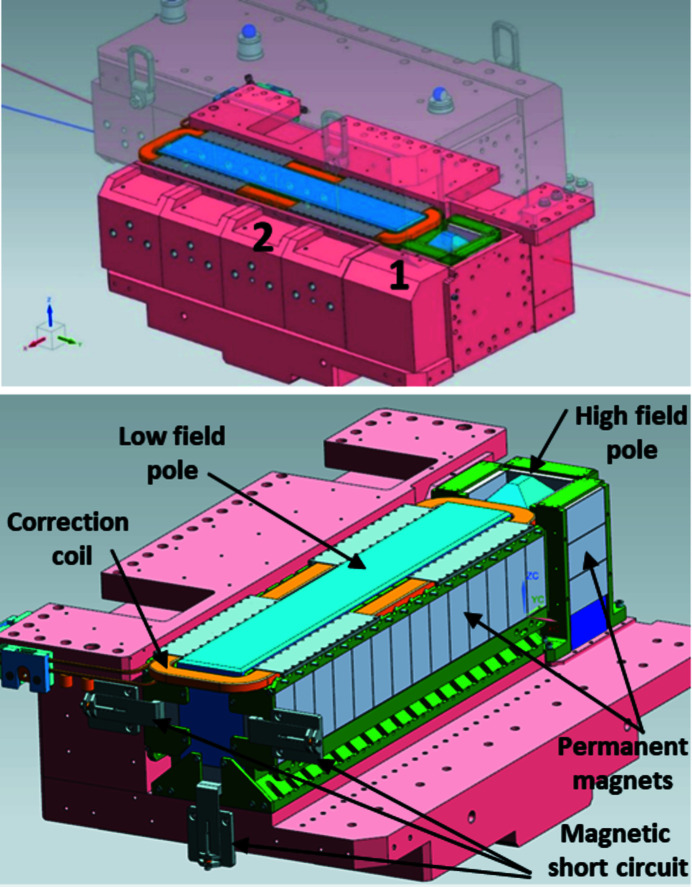
View of the high (1) and low (2) magnetic field parts of the Superbend, with (top) and without (bottom) the yoke.

**Figure 5:**
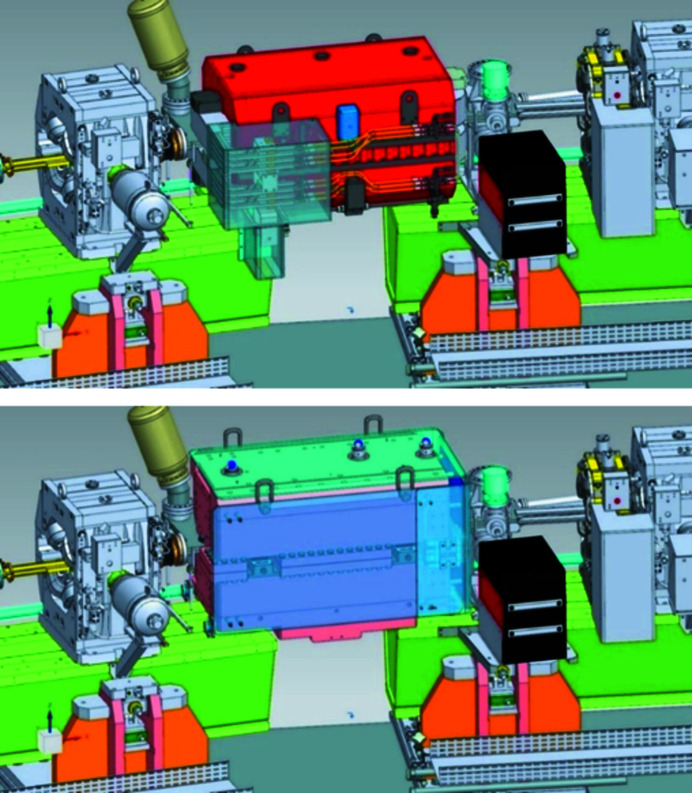
(Top) View from inside the storage ring of the standard electromagnetic dipole. (Bottom) Same view with the Superbend dipole.

The basic mechanical concept of the Superbend dipole consists of keeping as much as possible the standard electromagnetic dipole configuration (Filhol, 2004 ▸), in terms of mass and mass center, and to keep unchanged the frequency range of the vibration modes and all the interfaces existing around the dipole. The axis of the ROCK photon beam must remain as close as possible to its initial position leading to a local increase of the magnetic field, just at the photon source point location. Then the Superbend consists of two half-yokes, each including a pole called ‘low magnetic field’ and a pole called ‘high magnetic field’ (Fig. 4 ▸). To remain compatible with an *in situ* baking of the vacuum vessel, without heating the PMs, the Superbend dipole has a C-shape, open on the outside of the ring, that allows it to be shifted on the side and away from any heating source (Fig. 5 ▸). The deformation of the C-shaped yoke at the poles is estimated to be 0.020 mm, following a first calculation made during the design of the Superbend. Note that, for the assembly of the PMs, benefits from experience of the Sirius new Brazilian synchrotron light source were taken (Citadini *et al.*, 2018 ▸), especially with regard to the conception of dedicated tools, which was a key point for the success of the project.

### 3.3. Assembly of PMs

**Figure 6:**
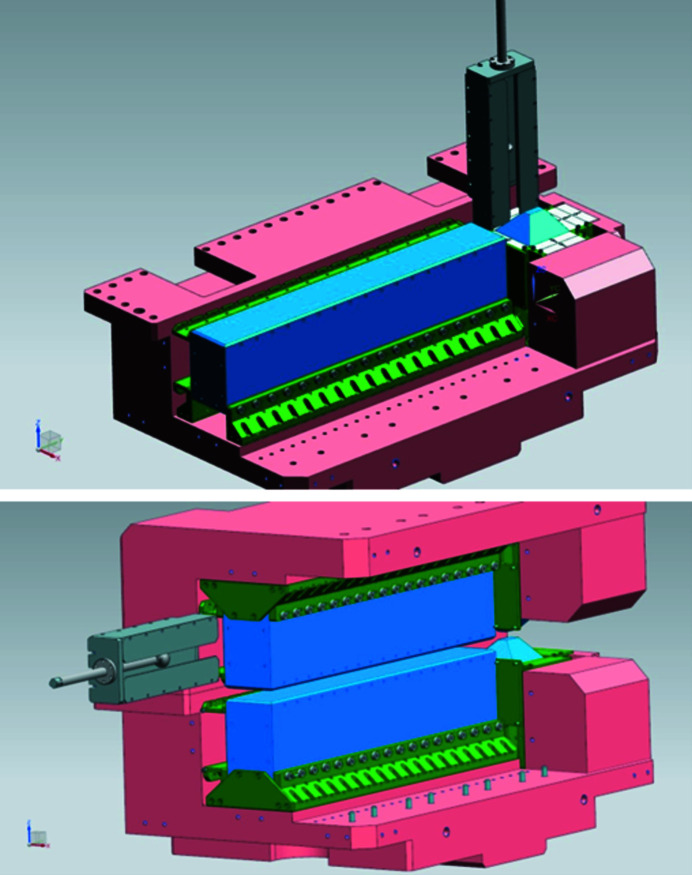
The tools (in gray) used for the installation of PMs around the high field poles (top) and low field poles (bottom).

The PMs were assembled in pairs before being positioned around the poles. All 234 PMs were first measured individually in order to be able to sort them and magnetically optimize the assembly of the magnet pairs. In particular, the angle error was minimized, or even canceled, for each pair of magnets. No automatic sorting program was used – only manual sorting was carried out using an EXCEL file. The magnetic calculations showed that attraction forces reach 8000 daN between the poles and 26000 daN between the two half-yokes. These forces are too strong to allow assembly of the PMs in the two separate half-yokes before assembly of the two half-yokes. First, after assembling the pole holding mechanism in each of the two half-yokes, PMs were assembled around the two ‘high-field’ poles using appropriate tools (Fig. 6 ▸), designed and built at SOLEIL. Then, the two half-yokes were assembled, using dedicated tools, to take into account that the attraction force between the two high-field poles, equipped with their PMs, is 800 daN. The lower yoke was fixed on the assembly table, and it was necessary to design and build special fixtures to rigidify the system and prevent the upper yoke from swinging when approaching the lower yoke. The vertical gap between the upper and lower poles was then fine-tuned to the nominal value. Once the two half-yokes were assembled, it was possible to insert the PMs around the two ‘low-field’ poles using dedicated tools (Fig. 6 ▸). The challenge was to correctly position the first PMs at the end of the guide (at about 0.6 m from the entrance) to avoid some blocking because removing an inserted PM was impossible due to the magnetic attraction forces involved.

### 3.4. Magnetic measurements

#### 3.4.1. Measurements of PM blocks

**Figure 7:**
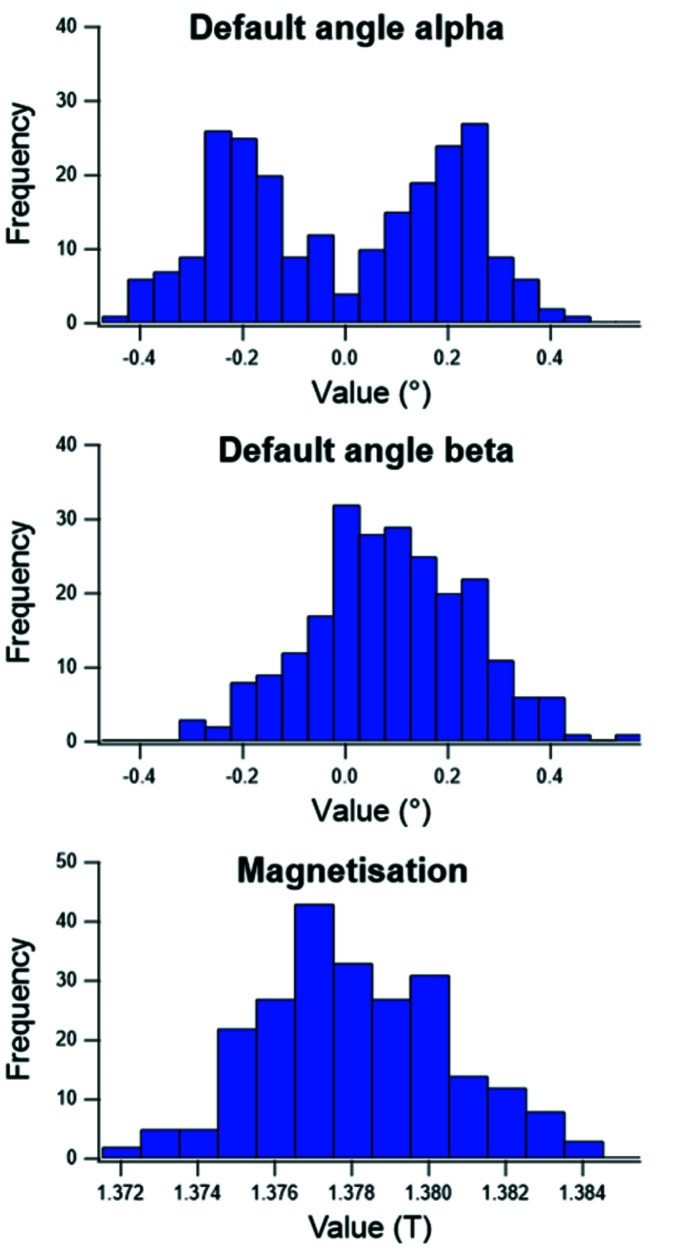
Distribution of the measured angular defect and magnetization for all the PM blocks.

As mentioned previously, each PM block is made of two smaller PM blocks. To decide which small blocks were associated, all the small PM blocks were measured by means of a Helmholtz coil. This measurement gives the three components of the magnetization of each small block. Fig. 7 ▸ shows the data for all the small blocks. The overall standard deviations of the α angle, β angle and magnetization are 0.22°, 0.16° and 2 mT, respectively.

#### 3.4.2. Measurement of the magnetic field by Hall probe

**Figure 8:**
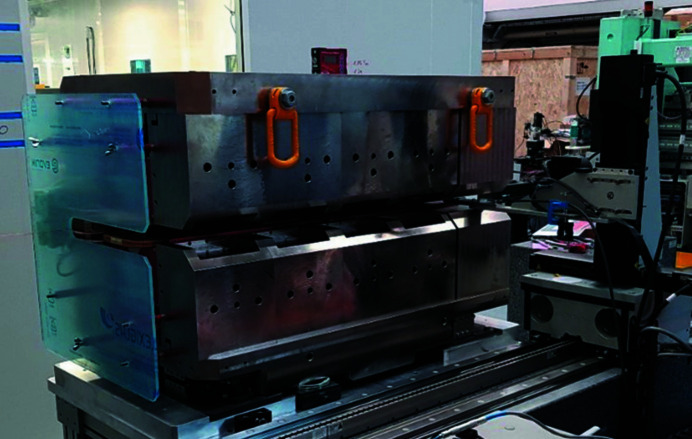
The PM Superbend installed on the Hall probe bench in the SOLEIL laboratory.

**Figure 9:**
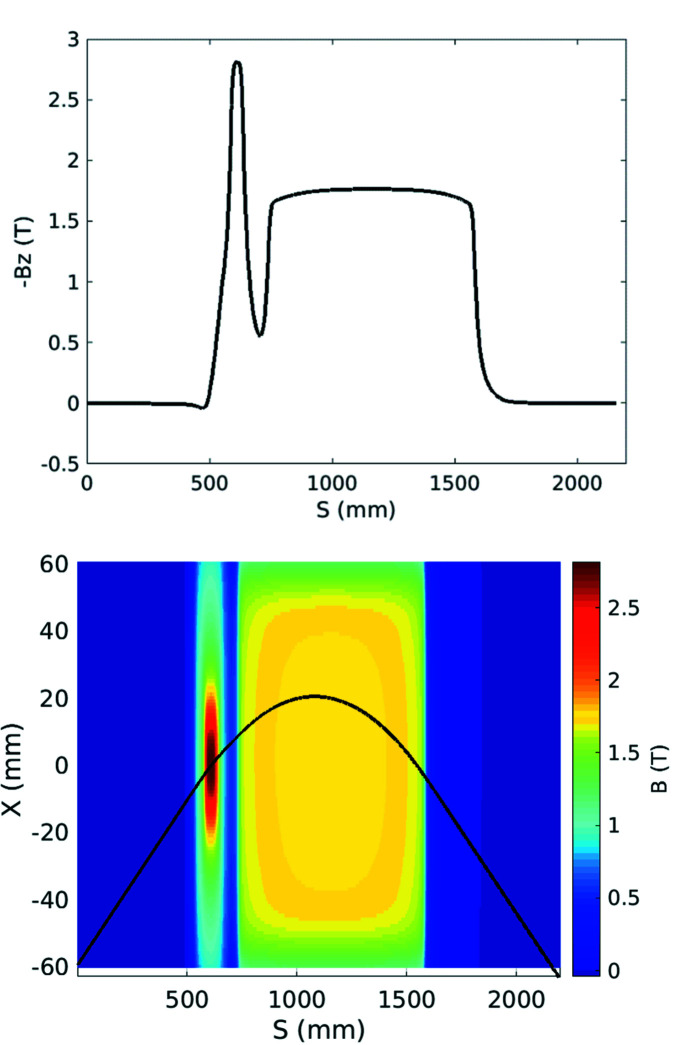
(Top) Variation of the magnetic field along the beam trajectory in the Superbend. (Bottom) Beam trajectory (in black) deduced from the measured 2D magnetic field mapping (in colors).

The dipole has been installed on a Hall probe bench (Fig. 8 ▸). This bench provides a three-axis translation (3500 mm × 250 mm × 250 mm). All the translation axes are driven by an XPS Newport controller. The Hall voltages are read by three Keithley voltmeters. All these devices are controlled by *IgorPro* software together with a Tango interface. The Hall probe is an ultra-low-noise system developed and calibrated up to a 4 T field by Senis (https://www.senis.swiss). The magnetic measurement consists of a 2D field map in the median plane of the dipole. An iterative process has been used to adjust the total field integral to the one corresponding to the standard 11.25° deviation (1.793 T m), taking into account the curvature of the electron trajectory. The process consisted of two steps: (i) adjustment of the stainless-steel thickness inserted between the lower and the upper yokes, and (ii) a fine tuning with the magnetic short circuit. The final 2D field map and the variation of the field along the beam trajectory are shown in Fig. 9 ▸.

#### 3.4.3. Measurement of the field integral with a single stretch wire

To verify the accuracy of the Hall probe magnetic measurement, the dipole has been installed on a single stretched wire bench [manufactured by the ESRF (Le Bec *et al.*, 2012 ▸)] in the SOLEIL magnetic measurement laboratory. This bench is designed to compute the harmonic content of a magnetic field from the induced voltage generated when the wire is describing a circular trajectory in the gap of the magnet. As the wire is stretched, the integrated field is measured over a straight line, and can be compared with the integrated field measured with the Hall probe. The difference between the two measurements was less than 0.1%, thus giving real confidence for the installation of the dipole in the storage ring. As described in the next subsection, this small difference can easily be explained by the change in temperature between the two measurements.

#### 3.4.4. Effect of the temperature

**Figure 10:**
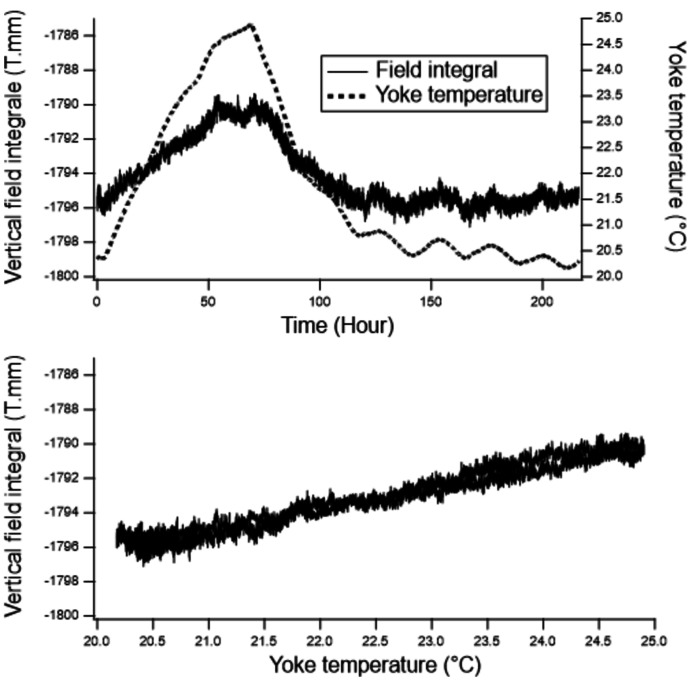
Variation of the field integral during testing of the sensitivity to the yoke temperature. (Top) Versus time. (Bottom) Versus yoke temperature.

To anticipate temperature variations in the storage ring tunnel during operation, it was important to measure the sensitivity of the integrated field to the temperature of the PM. Integrated field measurements versus temperature were performed on the single stretch wire bench, and in addition a Hall probe was fixed in the gap of the LFP to measure the local vertical field. Over a period of ten days, the integrated field, the field of the LFP and the temperature of the yoke were measured and recorded. Furthermore, the setting point of the room temperature of the lab was increased by 5°C and it took about two days for the dipole yoke’s temperature to increase as well. After two days, the setting point was set back to its normal value. Fig. 10 ▸ shows the variation of the field integral during these tests. The temperature effect on the field integral is 0.07% per °C which is a bit lower than the sensitivity of the PM remanent field versus the temperature. The difference is due to the saturation of the poles.

### 3.5. Installation

To allow installation of the Superbend dipole in the storage ring and to allow it to be shifted on its side in case of vacuum vessel baking, a dedicated mechanical support equipped with a system allowing adjustment of its horizontal and vertical positions was designed and built. This equipment has the ability to move a mass of more than 3500 kg over a lateral distance of 600 mm above cable trays and other utilities. The poles of the Superbend dipole are so close to the vacuum vessel that it was mandatory to lift the dipole vertically by about 0.4 mm above the beam axis before translation of the dipole. During all stages of placement and extraction, it was obligatory to have a look at the alignment devices to permanently check the dipole position with respect to the vacuum vessel. Finally, the Superbend was set up leaving only 0.2 mm of vertical space between the vacuum vessel and the high field magnetic pole. Note that the support was also used to extract the lower yoke of the standard electromagnetic dipole allowing the new vacuum chamber to remain unmoved and thus avoiding bake-out and the vacuum conditioning phases, thus saving almost two weeks in the planning.

## 4. Design and construction of the new vacuum vessel

### 4.1. Mechanical design

**Figure 11:**
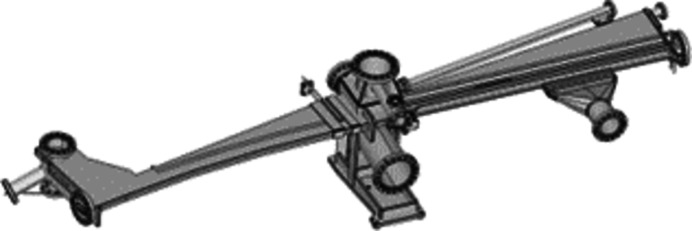
Original vacuum vessel installed in the previous ROCK electromagnetic dipole.

**Figure 12:**
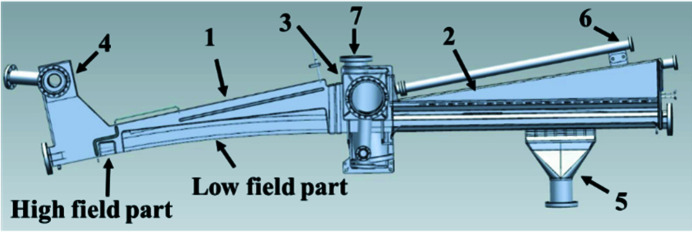
Top view layout of the new Superbend vacuum vessel. (1) Dipole part; (2) quadrupole part; (3) crotch box; (4) pumping sub-assembly; (5) quadrupole pumping port; (6) beamline outlet; (7) crotch absorber flange.

**Figure 13:**
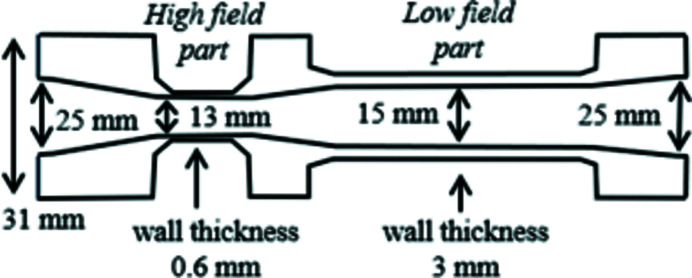
Layout of the transverse profile of the new vacuum vessel. The wall thickness of the high field part was reduced to 0.6 mm thanks to the small surface of the pole (only ∼50 mm × 50 mm). All the transition taper angles were fixed to 0.02 rad.

The ‘as built at the beginning’ storage ring includes, among many vacuum vessels, 32 non-magnetic stainless-steel dipole vacuum vessels, with slight differences according to location on the ring itself. For this purpose, dipole vacuum vessels were designed in three parts: the first one is a common part designed to be received inside the dipole magnet strictly speaking, the second part comprises a box destined to house the crotch absorber and to bind with the third part (called quadrupole part) which can be different in terms of length, depending on the type of quadrupole and sextupole magnets that follow the dipole and on the distance between them. The vacuum chamber installed in the original ROCK electromagnetic dipole is shown in Fig. 11 ▸. As the vertical gap required between the poles of the new Superbend dipole was lower than those of the standard electromagnetic dipoles, it was not possible to keep the common first dipole part of the vacuum vessels as designed. As the quadrupole part should be identical, the original design was kept (Filhol, 2004 ▸; Level *et al.*, 2003 ▸, 2005 ▸; Filhol *et al.*, 2006 ▸) and only the dipole part was adapted to the new gap requirement (Fig. 12 ▸). Moreover, one of the design constraints for the Superbend implantation was to keep the identical crotch absorber, confirming the design of the new vacuum vessels. Finally, the new vacuum vessels had to accommodate the new dipole magnet gap, keeping the same crotch absorber in the same housing box that binds with the standard quadrupole part. As well as the difficulty in accommodating a very low vertical gap for the 2.8 T magnetic bore, the complementary ‘low-field’ part of the Superbend dipole also needed a lower gap than the actual one because a magnetic field slightly higher than 1.7 T was needed to reach the nominal total field integral. Nevertheless, the dipole part was designed on a similar principle as the actual one (Filhol, 2004 ▸; Level *et al.*, 2003 ▸, 2005 ▸; Filhol *et al.*, 2006 ▸), keeping the same manufacturing scheme in two half shelves, assembled with an electron beam welding in the median plane. This allows the shape to be adapted to both magnetic gaps and electron beam path (Fig. 13 ▸), keeping a good mechanical rigidity and strength, and keeping the non-magnetic properties of the 316LN stainless-steel raw material. In addition, the transition between the different gaps along the chamber was designed to minimize heating due to the interaction of the electron beam with the walls of the vacuum chamber (impedance effect).

### 4.2. Construction

**Figure 14:**
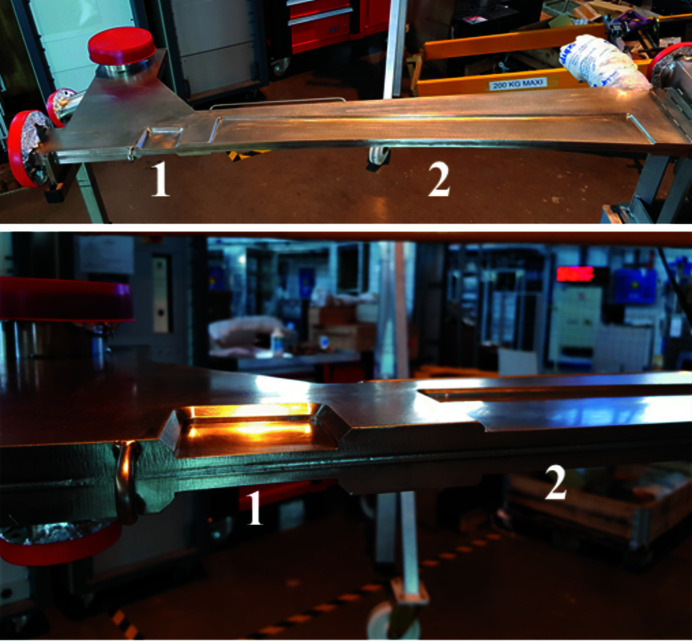
(Top) high (1) and low (2) field gap footprint on the dipole part of the new vacuum vessel. (Bottom) Zoom of the transition from the high (1) to low (2) field parts.

For the construction of the non-magnetic stainless-steel vacuum vessels, it was quite natural to entrust the same manufacturer that realized the series of 37 original SOLEIL dipole vacuum vessels. Back in the 2004–2006 period, many problems were addressed and solved for this procurement. Thus, tools to achieve mechanical tolerances were kept by the manufacturer, and already reused in 2010–2011 for the manufacturing of another specific vacuum vessel. Knowing the minimum delay of nine months, easily extendable to one year, the manufacturing of a the new Superbend vacuum vessel was launched early in May 2019. The final vacuum vessel (Fig. 14 ▸) was delivered to SOLEIL in December 2020, with a more extended planning than foreseen, partially because of the Covid-19 pandemic and because the dipole part appeared to be more difficult to machine than predicted.

### 4.3. Installation

**Figure 15:**
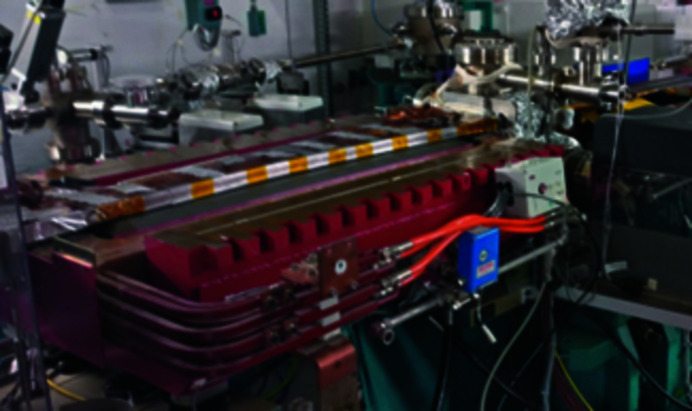
The Superbend vacuum vessel installed temporarily inside the standard electromagnetic dipole and equipped with heating films. The upper yoke of the electromagnetic dipole is not yet back in place in the photograph.

**Figure 16:**
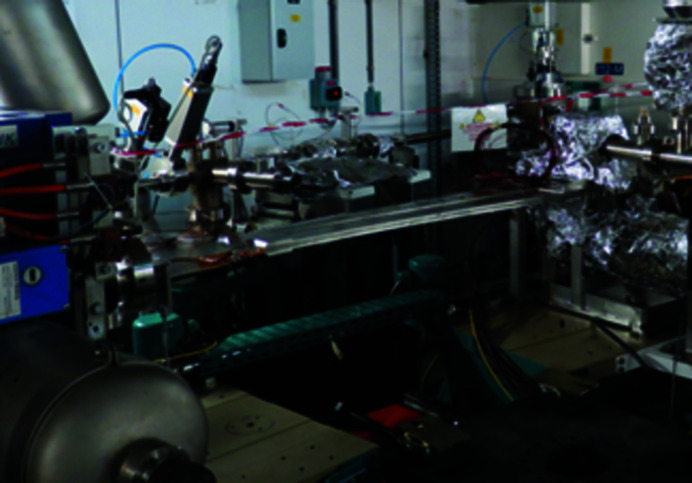
The Superbend vacuum vessel installed in the final configuration, ready to receive the Superbend.

The vacuum vessel was installed in the storage ring during the winter-long technical shutdown of January 2021, by only replacing the standard vacuum vessel. The new vessel was installed first inside the standard electromagnetic dipole as the Superbend dipole was not ready to be installed at that moment (Fig. 15 ▸). The vessel was baked-out *in situ* as is usually done at SOLEIL. Because of the notches made on the dipole part of the new vessel to accommodate the high and low field poles, the Superbend vessel could not be equipped with Kapton heating films for *in situ* bake-out, so the notches were temporary filled in with aluminium wedges to glue heating thin films on the vessel. In August 2021, before the final installation of the Superbend, the temporary *in situ* bake-out system was removed from the dipole part of the vacuum vessel (Fig. 16 ▸). As the magnetic properties of the PMs deteriorate for temperatures higher than 120°C, if some bake-out is required in the future then the Superbend should be extracted and placed on the support, beside the vacuum vessel, and conventional heating tapes should be used.

## 5. Effect of the new dipole on electron beam dynamics

### 5.1. Modeling

**Figure 17:**
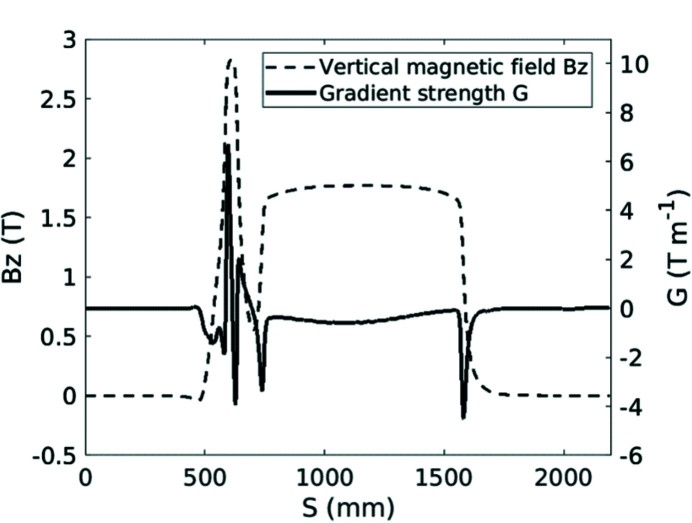
Variation of the quadrupolar component (solid line) along the beam trajectory in the Superbend. The magnetic field (dotted line) is plotted as a reference.

**Figure 18:**
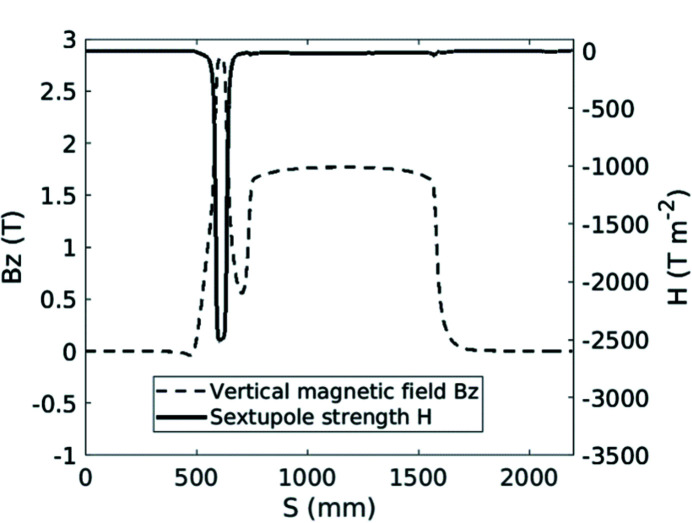
Longitudinal variation of the sextupolar component (solid line) generated mainly by the high field of the Superbend. The magnetic field (dotted line) is plotted as a reference.

**Figure 19:**
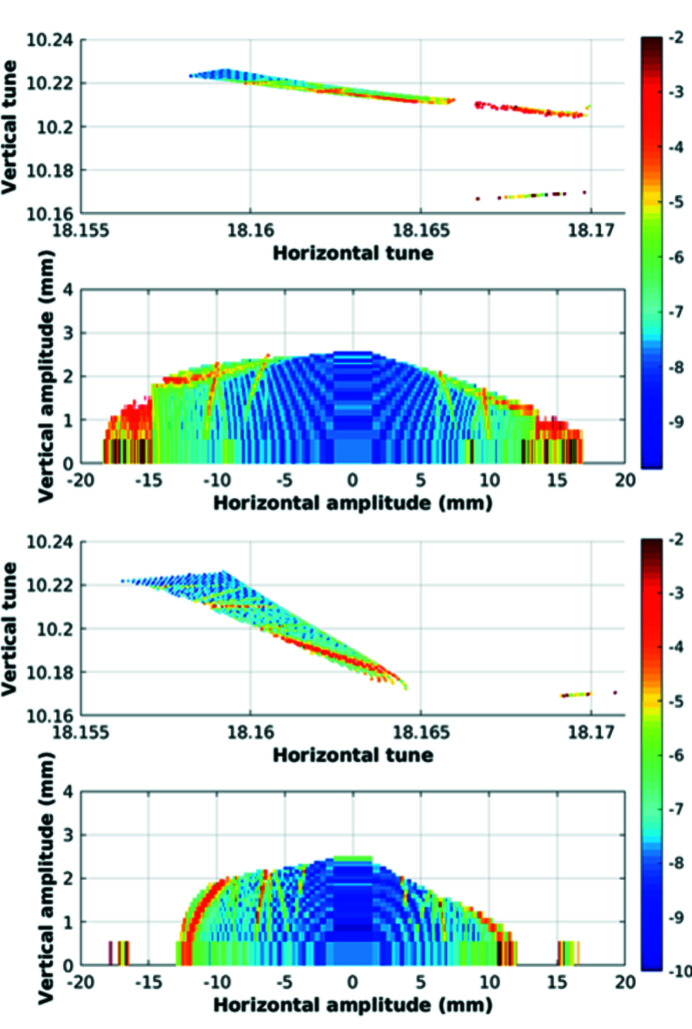
On-momentum dynamic aperture and corresponding frequency map calculated at the injection point. (Top) Without Superbend. (Bottom) With Superbend after compensating for the focusing effect. Simulations were performed with the SOLEIL version of the 6D true symplectic *TRACY* tracking code. The color bar indicates the tune diffusion rate after 2 × 1026 turns.

The lattice model of the SOLEIL storage ring includes all the magnet multipolar components, the fringing fields of the dipoles and quadrupoles and the horizontal and vertical physical apertures at each element. As the model is considered to be robust owing to comparisons with experimental results, it was mandatory to have a precise modeling of the Superbend field to anticipate linear and nonlinear effects and to compensate for them. Then the 2D measured field mapping was used to evaluate all the multipolar components generated by the Superbend field, from quadrupolar to dodecapolar ones, without neglecting the longitudinal variation of the magnetic field, due to the passage from the ‘high-field’ region to the ‘low-field’ region, that generates some focusing. First the trajectory in the Superbend was deduced from the magnetic field mapping (Fig. 9 ▸). Then the multipolar components were calculated around the beam trajectory as a function of the longitudinal position. The longitudinal variation of the quadrupolar component (Fig. 17 ▸) led to splitting of the Superbend in seven parts of different length in the model, each part including all the corresponding integrated multipolar components. Another important component is the sextupolar component generated mainly by the high field (Fig. 18 ▸). Due to a high value of the vertical beta function at the Superbend location, the vertical beta-beating generated by the focusing of the Superbend reached 15% and was compensated using four quadrupoles located upstream and three quadrupoles located downstream of the Superbend. Although no significant effect on the Touschek lifetime was predicted, the on-momentum dynamic aperture was significantly reduced for negative horizontal amplitudes (Fig. 19 ▸) thus inducing a reduction of the injection efficiency.

### 5.2. Commissioning of the new vacuum vessel and the new dipole

**Figure 20:**
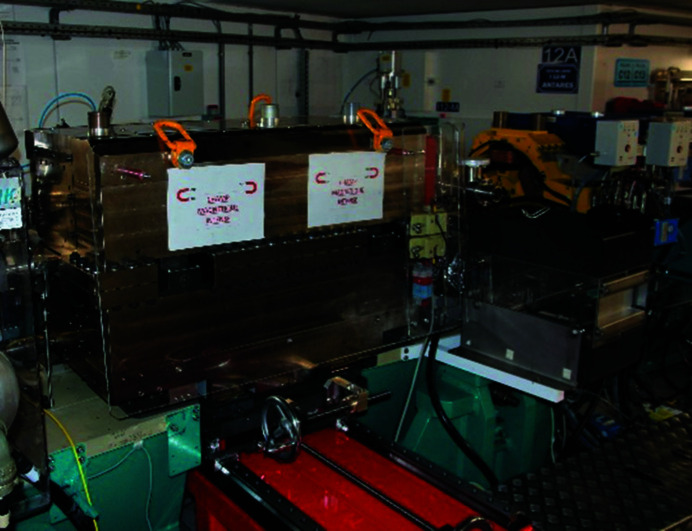
The Superbend installed in its place in the storage ring tunnel. In red, the frame equipment dedicated to extracting the dipole if a vacuum vessel bake-out must be performed.

**Figure 21:**
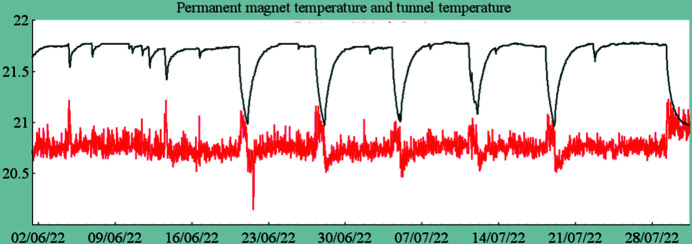
Example of the temperature variation in the storage ring tunnel during operation. (Red) Air tunnel temperature. (Black) PM temperature. Temperatures on the vertical axis are given in °C.

In January 2021, the storage ring was restarted after the winter shutdown with the new vacuum vessel installed in the standard dipole as the Superbend was not yet ready. This allowed the limitations due to the reduced aperture of the vacuum vessel to be distinguished from those imposed by the new magnetic field. Despite a high pressure measured with the first stored electron beam of small intensity, the vacuum conditioning was very efficient, and the high current of 500 mA could be delivered to the users four days later. The large magnetic gap of the standard electromagnetic dipole allowed many thermocouples to be installed along the new vacuum vessel. As expected from the optimized profile, the analysis of the measured temperatures showed similar behavior to that measured with the old vacuum vessel, without any hot point. The beam loss monitors, installed upstream and downstream of the new vacuum vessel, showed that the reduction of the vertical physical aperture did not localize the losses at this place. The vertical scrapers, set at their nominal position, remain the smallest physical aperture in the whole ring. Further experiments performed by shifting the electron beam vertically in the new vacuum vessel showed that the minimum total vertical aperture was close to the expected value of 12 mm, thanks to very accurate alignment of the new vacuum vessel. The ROCK beamline found the photon beam right where it was before the installation of the new vacuum vessel, as expected. In May 2021, the blades of the X-Beam Position Monitor (XBPM) installed on the ROCK beamline were changed to anticipate the increased power of the photon beam generated by the Superbend. In August 2021, the Superbend was installed in the storage ring tunnel (Fig. 20 ▸) and on 25 August the first electron beam was stored in the presence of the Superbend. The temperature measured in the tunnel at the Superbend location was very close to that measured during the magnetic measurements, and, as expected, no field integral correction was required. The electron trajectory was adjusted using only a few tenths of an ampere on the closest dipolar corrector power supplies. This was followed by a series of experiments to characterize and optimize the performance of the storage ring. As expected, the vertical chromaticity was increased by one unit due to the sextupolar component generated by the high field roll-off. Symmetrization of the optics has shown that the quadrupolar component of the Superbend was larger than expected. This can be explained by the fact that the reference axis of the new dipole, registered on the magnetic measurement bench, may be slightly shifted in the horizontal (0.2 mm) at the location of the high field where there is a strong sextupolar component. Indeed, it was difficult to center the magnetic axis in both the high and low field regions. The high field sextupolar component also affects the injection efficiency and the beam lifetime. For the bare machine without insertion devices, the injection efficiency is reduced from 80 to 65%, and, when insertion device fields are varying during the operation, the sensitivity to the betatron tunes has been increased, making the operation less easy than before the installation of the Superbend. Finally, in daily operation, the injection efficiency and the beam lifetime were maintained at around 60% and 8 h, respectively (https://www.synchrotron-soleil.fr/fr/faisceau). Despite the expected decreases in injection efficiency and beam lifetime, the electron beam characteristics at the source points were not changed, neither in terms of transverse dimensions nor transverse position. For the standard mode of operation, the use of a multi-objective genetic algorithm such as MOGA-BMAD (Ehrlichman, 2016 ▸) has provided a new set of sextupoles that compensates for the non-linear effects of the Superbend, and since May 2022 the operating performance has returned to pre-Superbend values in terms of injection efficiency and electron beam lifetime. To measure the temperature of the Superbend dipole, temperature probes were glued onto some of the PMs. Fig. 21 ▸ shows the temperature variation during a period of two months. The temperature increases from 21°C to 21.5°C in the presence of the electron beam. This increase is due to the proximity of the vacuum chamber which is heated by synchrotron radiation. The small effect of the temperature on the integrated field is seen on the electron closed orbit and is compensated by the Slow Orbit Feedback and, as expected, only the two dipolar correctors close to the Superbend dipole (located in the upstream and downstream sextupoles) are slightly varying. There are no requirements to use the Superbend correction coils.

A disappointing consequence concerns the so-called ‘low-alpha’ mode which is used in the operation a few weeks per year. The effect on the beam dynamics of the high field sextupolar component is amplified when this optics is used because the value of the vertical beta function at the Superbend location is three times larger than the standard one. Even the use of MOGA-BMAD did not allow a return to acceptable beam dynamics and the experimental tests led to a very poor injection efficiency. This mode of operation has now been abandoned.

## 6. First results on the ROCK photon beamline

**Figure 22:**
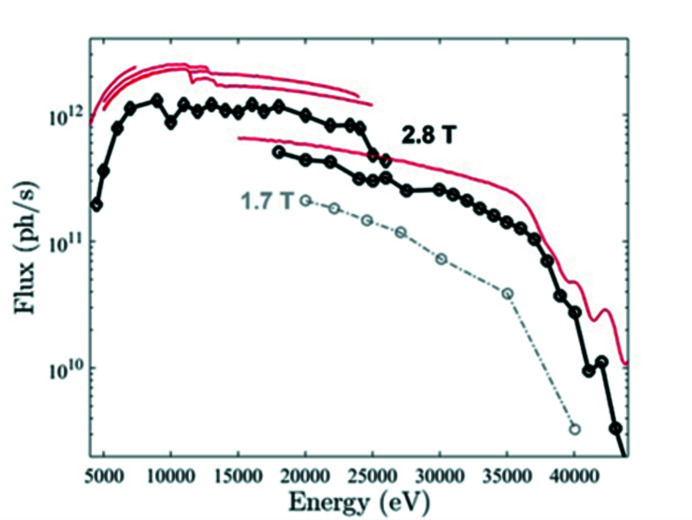
Black lines are the flux of the monochromatic beam measured at the sample position for a full spot size, plotted as a function of X-ray energy for an electron beam current of 500 mA and 2.8 T source. Diamonds are related to measurement made with the Si(111) monochromator whereas circles relate to measurements carried out with the Si(220) monochromator. For comparison purposes, the flux measured at the sample position for the 1.7 T source and Si(220) monochromator (gray dotted lines) is reported as well as the flux calculated for perfect optics using the 2.8 T Superbend (red lines).

**Figure 23:**
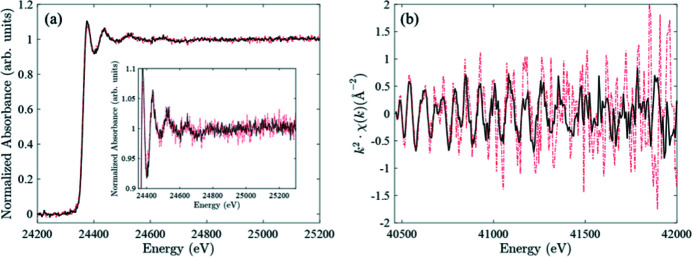
(*a*) Pd *K*-edge normalized absorption spectra recorded for a 1 wt% Pd loaded catalyst supported on CeO_2_ (measurement in transmission in 5 s using the 2.8 T source and 12.5 s using the 1.7 T source). A zoom on the upper part is added. (*b*) Ce *K*-edge *k* ^2^χ(*k*) EXAFS spectra recorded on a CeO_2_ pellet (measurement in transmission in 5 s). Dotted red lines correspond to measurements made with the 1.7 T bending magnet source and black lines to those made with the 2.8 T Superbend.

It is noteworthy that the power density received by the first mirror of ROCK and used for the design of its cooling system together with the radiation requirements of shielded hutches was calculated at the early stage of the beamline design with a source of 3 T as a target for an upgrade of the beamline a few years after starting the operation. A liquid-nitro­gen-cryo-cooled Quick-EXAFS monochromator, mechanically equivalent to the two already used water-cooled monochromators designed and built at SOLEIL for first operation at the SAMBA beamline (Fonda *et al.*, 2012 ▸), was nevertheless installed at the beamline in 2020 to remove the distortions on the Si(220) channel-cut crystal induced by the significant increase of beam power load when passing from the 1.7 T to the 2.8 T source (La Fontaine *et al.*, 2020 ▸). As the position of the ROCK source point was kept unchanged, no realignment of the optics was necessary, and on 27 August, two days after the first electron beam, the first photon flux curves delivered by the newly installed Superbend were measured using the water-cooled Si(111) and cryo-cooled Si(220) monochromators. Fig. 22 ▸ displays the experimental photon flux between 5 and 43 keV for different grazing incidences of the pink beam delivered by the first mirror aligned at 2.25 mrad on the second- and third-harmonic rejection mirrors located between the monochromators in the optics hutch (Briois *et al.*, 2016 ▸). The measured flux curves are compared with those calculated at the sample positions for perfect optics with the 2.8 T Superbend source and those measured using the 1.7 T former ROCK source with the Si(220) monochromator. Calculations and measurements relate to a 500 mA beam current. A striking increase of the flux values above 20 keV with the 2.8 T source has been measured and agrees with the calculations. Photon fluxes higher than 10^11^ photons s^−1^ are achieved up to 35 keV instead of 25 keV with the 1.7 T source. These higher photon fluxes have already benefited several user experiments and allow for:

(i) faster measurements keeping absorbing element loading and S/N with nearly equivalent quality [Fig. 23 ▸(*a*)],

(ii) lower detection limit of the absorbing element at equivalent time resolution and S/N (not shown),

(iii) better S/N of spectra everything else being equal [Fig. 23 ▸(*b*)].

**Figure 24:**
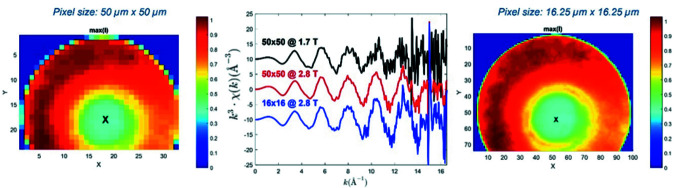
Comparison of *k* ^3^χ(*k*) EXAFS spectra extracted from full-field TXM images recorded on an alumina cylindrical extrudate impregnated with a 1 *M* Mo solution with the 1.7 T source and the 2.8 T source. The spectra were extracted from the pixel at the center of the extrudate but considering different binnings of the camera pixel image. The image on the left (using the 1.7 T source) has been binned to reach a pixel size of 50 µm × 50 µm whereas at the right (using the 2.8 T source) the pixel size is 16.25 µm × 16.25 µm (Barata, 2023 ▸).

Furthermore, in the case of full-field transmission X-ray microscopy imaging with micrometer spatial resolution recently implemented at the beamline (Fonda *et al.*, 2012 ▸), even the increase by only a factor of two of photons at the Mo *K*-edge (20 keV) has been efficient for improving the quality of the EXAFS spectra extracted on pixels of binned images obtained by the average of 120 stacked energy-cubes. It is clearly showed in Fig. 24 ▸ that:

(i) at equivalent spatial resolution, the S/N of the EXAFS spectrum extracted on a 50 µm × 50 µm pixel image is better at high *k* value for the data recorded with the 2.8 T source than for those recorded with the 1.7 T source,

(ii) equivalent S/N data are extracted from images binned at a resulting pixel size of 16.25 µm × 16.25 µm for the data recorded with the 2.8 T source compared with those extracted from images binned at a resulting pixel size of 50 µm × 50 µm for those recorded with the 1.7 T source.

## 7. Conclusion

The construction of the ROCK Superbend permanent-magnet dipole has allowed a lot of experience to be gained with permanent magnets that will be very useful for the SOLEIL Upgrade (https://www.synchrotronsoleil.fr/en/news/conceptual-design-report-soleil-upgrade) as the intensive use of permanent magnets is foreseen. The goal of this innovative project was achieved by overcoming unforeseen difficulties, thanks to very reliable magnetic calculations and after designing and building tools perfectly adapted to each stage of the assembly. The temperature stabilization of the magnetic measurement hall, to a value of temperature close to that of the storage ring tunnel, provided the exact adjustment of the field integral avoiding using some correction system. In addition, this allowed sufficiently accurate measurements to be obtained to perform a very reliable modeling of the beam dynamics and to anticipate the significant nonlinear effect of the strong sextupolar component generated by the high field part of the dipole. The Superbend has been in operation at SOLEIL since 7 September 2021, and the nominal performance is now restored in terms of injection efficiency and electron beam lifetime. There is no need to use the correction coils – the effect of the PM temperature on the integrated field is well managed by the Slow Orbit Feedback and the required corrector strengths to compensate this effect are a few microradians compared with the milliradian range capacity of the correctors. The installation of the Superbend was ‘transparent’ for operation of the other photon beamlines in terms of photon beam position and stability. The ROCK beamline benefits now from a photon source that is much better adapted to its research. The data quality recorded at the high energy range of the electromagnetic radiation delivered by the Superbend has been significantly improved leading to faster recordings, more sensitive characterization of diluted elements, and spatially more sensitive measurements for full-field transmission microscopy.

## Figures and Tables

**Table 1:** Main parameters of the Superbend and comparison between design and final values

Parameter	Design value	Final value
Total length (m)	1.26	1.26
Maximum field (T)	2.81	2.84
Integrated field (T m)	1.803	1.793
High field gap (mm)	16.10	16.01
Low field gap (mm)	23.00	23.20
Size of the high field pole tip (mm)	19.5 × 45	19.5 × 45
PM size (mm)	120 × 60 × 60	120 × 60 × 30
PM remanent field (T) / RMS value (T)	1.36 / 0.0055	1.38 / 0.0025
PM coercitive force (kA m^−1^)	−1060	−1080
PM RMS angular defect alpha (°)	1	0.22
PM RMS angular defect beta (°)	1	0.16
Correction coil power at 100 A (W)	600	600
